# Exploring the Role of Amitriptyline in Modulating Gag Reflex Sensitivity

**DOI:** 10.5812/ijpr-160912

**Published:** 2025-10-15

**Authors:** Mehdi Modiri, Reyhaneh Shoorgashti, Farnaz Haji Fattahi, Simin Lesan

**Affiliations:** 1 Faculty of Dentistry, Dental Branch, Islamic Azad University of Medical Sciences, Tehran, Iran; 2 Department of Oral Medicine, TeMS.C., Islamic Azad University, Tehran, Iran

**Keywords:** Gagging, Amitriptyline, Lidocaine, Local Anesthetics

## Abstract

**Background:**

The gag reflex serves as an essential protective airway mechanism but can significantly interfere with dental care, affecting up to 44% of patients and leading to treatment avoidance in severe cases.

**Objectives:**

To evaluate the effect of the topical application of an amitriptyline solution on reducing the gag reflex intensity, measured by the Gag Trigger Point Index (GTPI).

**Methods:**

In this randomized single-blind clinical trial, 48 participants with a GTPI score higher than two were divided into amitriptyline (treatment) and lidocaine (control) groups. In the amitriptyline group, 75 mg of amitriptyline tablets were dissolved in 5 milliliters of distilled water (15 mg/mL) and gargled for one minute by the participants. Then, the GTPI was examined after 10 minutes. In the lidocaine group, four puffs of 10% lidocaine spray were applied to the target areas of the oral mucosa, and the GTPI was measured five minutes later. The taste and smell of both medications were assessed using a self-report questionnaire to measure patient satisfaction. The data were analyzed using SPSS version 22.

**Results:**

In both the lidocaine and amitriptyline groups, GTPI levels demonstrated significant decreases. The lidocaine group showed a change from 4.46 to 2.42 (P < 0.001), and the amitriptyline group showed a change from 4.04 to 1.29 (P < 0.001). The reflex change rate was -2.75 in the amitriptyline group and -2.04 in the lidocaine group. When comparing the groups, no statistically significant differences were observed in the extent of gag reflex reduction or in participants’ perception of taste and smell (P > 0.05).

**Conclusions:**

Amitriptyline can be considered a potential alternative to lidocaine spray in gag reflex management, particularly in lidocaine-intolerant patients. Further studies are needed to confirm long-term safety and determine the local versus systemic pharmacological effects.

## 1. Background

The gag reflex functions as a defensive mechanism that safeguards the respiratory tracts from the aspiration of foreign objects, with its intensity and triggers exhibiting considerable variability among individuals (1, 2). This reflex often presents a clinical obstacle, especially during procedures such as dental treatments and endoscopy (3-6). Reports suggest that approximately 8.2% of dental patients experience gagging issues, with the incidence rising to around 44% in those undergoing denture fittings (7, 8).

An excessively sensitive gag reflex arises from a combination of physiological and psychological mechanisms (9, 10). Overactive sensory input from oral tissues, transmitted via cranial nerves to the brainstem gag center, can trigger strong involuntary responses even with minor stimulation (11, 12). Anxiety, past negative dental experiences, and conditioned responses further heighten the reflex, while non-tactile stimuli such as dental sounds or odors may also provoke gagging. Medical factors like gastroesophageal reflux or neurological disorders can exacerbate sensitivity (11). Collectively, these mechanisms contribute to dental avoidance, with gagging reported as a reason for up to 20% of missed dental visits, ultimately leading to compromised oral health and tooth loss (7).

Various techniques have been explored to manage the gag reflex, but a universally effective method remains elusive. Behavioral techniques, topical anesthetics such as lidocaine, pharmacologic sedation, acupuncture, and acupressure are among the techniques studied for gag reflex control.

Amitriptyline, a tricyclic antidepressant, is approved by the U.S. Food and Drug Administration (FDA) for treating major depressive disorder (MDD) in adults (13, 14). Beyond its FDA-approved use, amitriptyline is employed off-label to address a range of conditions, such as anxiety, post-traumatic stress disorder, and chronic pain (15, 16). Boll et al. demonstrated a decrease in bronchial hyperresponsiveness, a reduction in eosinophil and characteristic TH2-lymphocyte numbers, along with diminished levels of IL-4 and IL-5 as a result of amitriptyline inhalation (17).

Amitriptyline shares structural and pharmacodynamic similarities with local anesthetics by blocking sodium channels in neuronal membranes, leading to decreased nerve excitability. When used topically, these effects can occur within minutes, independent of its antidepressant or anxiolytic systemic actions, which typically require prolonged use (11, 18-20).

There has been a dearth of research exploring the effects of amitriptyline on the gag reflex. Approaches like acupuncture and acupressure, whether accompanied by sedation or not, have yielded inconclusive evidence regarding their efficacy in reducing gagging. The prescription of medications such as lidocaine can pose challenges for patients due to its unpleasant taste, and documented cases of allergic reactions and overdoses have been reported (1, 21, 22). Considering its anti-allergic, anxiolytic, and analgesic properties, amitriptyline could be a potential treatment option for hypersensitivity of the gag reflex.

## 2. Objectives

The objective of this study is to assess the impact of amitriptyline on the gag reflex and compare its effectiveness with that of lidocaine.

## 3. Methods

**Figure 1.**
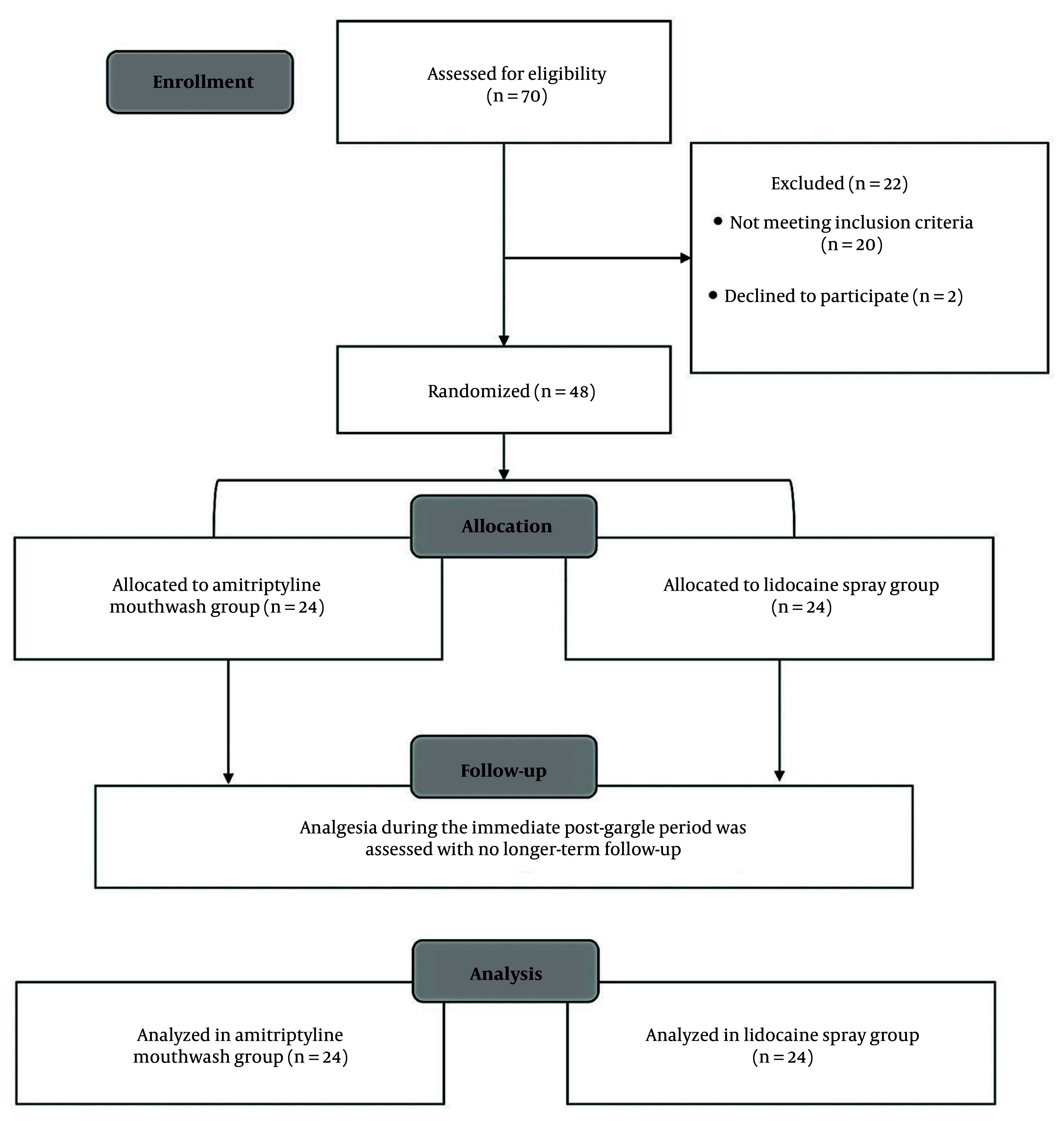
The Consolidated Standards of Reporting Trials (CONSORT) flowchart diagram

This randomized single-blind parallel clinical study was conducted in accordance with the Consolidated Standards of Reporting Trials (CONSORT) 2010 guidelines (Figure 1). 

### 3.1. Sample Size

A total of 24 subjects were assigned to each group, based on the study by Torabi et al. (23), with a power analysis targeting an effect size of 0.6, with α set at 0.05 and β at 0.2 to ensure sufficient statistical power. The aim was to detect a significant difference of at least 2 units.

### 3.2. Participants

A total of 70 volunteers were initially screened between January and December 2020 based on pre-defined inclusion and exclusion criteria. Ultimately, 48 individuals met the criteria and were assigned to groups using block randomization generated via Random Allocation Software version 2.0. Allocation sequences were concealed in opaque sealed envelopes opened only at intervention. All participants were dental students from the Islamic Azad University, Dental Branch, Tehran, Iran.

The first group received a 15 mg/mL amitriptyline mouthwash, and the second group served as the control and received a lidocaine spray. The amitriptyline mouthwash was prepared by dissolving 75 mg amitriptyline tablets in 5 mL distilled water (15 mg/mL = 1.5% w/v). The target was a brief, topical anesthetic effect over the soft palate and posterior pharyngeal wall, analogous in intent to high-concentration topical anesthetics used in dentistry, but delivered as a swish/gargle to coat the gag-trigger regions. Kakoei et al. (18) found that 0.1% mouthwash reduced mucositis pain without systemic adverse effects, indicating local efficacy at low concentration. Hasan et al. (24) showed 1% mouthwash/gel improved periodontal clinical and inflammatory parameters. Given the gag-trigger stimulation’s robust sensory input, we selected 1.5% w/v to ensure prompt surface anesthesia while restricting exposure to a single 1-minute gargle with complete expectoration.

The choice of a 1-minute gargling period was informed by prior studies evaluating topical anesthetic mouthwashes, including amitriptyline (18), benzydamine hydrochloride (23), and ibuprofen (20). Participants were instructed not to swallow; food/drink was withheld for 10 minutes to limit oropharyngeal clearance and potential ingestion (23).

### 3.3. Eligibility Criteria

Following the acquisition of informed consent, each participant's gag reflex sensitivity was assessed utilizing the Gag Trigger Point Index (GTPI). Individual scores were documented through a dedicated survey (23, 25). Inclusion criteria encompassed volunteers with a GTPI score exceeding 2, while exclusion criteria involved pregnancy, lactation, systemic disorders, motor neuron disease, or allergy to either medication, amitriptyline or lidocaine (25, 26).

### 3.4. Blinding

Participants were unaware of their group assignments. The allocation sequences were kept confidential in opaque sealed envelopes that were opened only at the intervention. Also, GTPI assessments were performed by outcome assessors who were not involved in drug preparation or administration and were blinded to treatment allocation. To reduce participant bias, amitriptyline mouthwash and lidocaine spray were kept in the same bottle and completely obscured with black-colored electrical tape. This precautionary step aimed to prevent participants from making assumptions about potential differences between the two medications prior to their application. Also, each participant underwent drug application and gag measurements separately and in isolation from others.

### 3.5. Study Procedure

All participants underwent examination sessions between 9 and 11 a.m., having consumed breakfast two hours prior. The stimulation of specific areas, as indicated in Table 1, was accomplished using a disposable wooden abslang, which made contact with the oral mucosa moving from anterior to posterior regions. To minimize any cognitive bias, participants were intentionally kept unaware of the methodology. These procedures were conducted by a dental student who had received training, operating under the supervision of an expert in oral and maxillofacial medicine, and another dental student was responsible for the selection, recording, and administration of the medication. The examinations were carried out while participants were seated in an upright position on a dedicated dental chair at the Department of Oral Medicine, Dental Branch, Islamic Azad University, Tehran, Iran.

**Table 1.** Gag Trigger Point Index Score Coded by the Location in the Oral Cavity Where the Gag Reflex Occurs (25)

Location of Gag Trigger Point	GTPI Score
**Posterior pharyngeal wall, no motor response**	0
**Posterior pharyngeal wall; motor response**	1
**Between posterior faucial pillars and posterior pharyngeal wall**	2
**Posterior faucial pillars**	3
**Between anterior faucial pillars and posterior faucial pillars**	4
**Anterior faucial pillars**	5
**Between second molars and anterior faucial pillars**	6
**Second molars**	7
**Internal cheek; center**	8

Abbreviation: GTPI, Gag Trigger Point Index.

In the amitriptyline mouthwash group, a solution was prepared by dissolving 75 mg of amitriptyline tablets (3 tablets of 25 mg amitriptyline, Iran Darou Pharmaceutical Company, Iran) in 5 mL of distilled water (Sepidaj Company, Iran). Participants gargled with the solution for 1 minute, and the GTPI test was repeated after a 10-minute interval. The oral mucosa was stimulated using a disposable wooden tongue depressor, applied from the anterior to the posterior regions. The procedure was identical to that performed at baseline, before the intervention (18, 23). For those in the lidocaine group, 4 puffs of lidocaine 10% spray (Iran Darou Pharmaceutical Company, Iran) were administered, and the GTPI test was conducted after a 5-minute interval (27). The testing procedure was conducted unilaterally (either on the right or left side) for each participant, both pre- and post-intervention. Patient satisfaction was evaluated using a qualitative self-report questionnaire. Participants could indicate their preferences by choosing from 'good', 'moderate', or 'weak' ratings, which were specifically related to the taste and odor of the medication used.

### 3.6. Data Analysis

The data collection for this study encompassed the use of a survey, observation, and clinical examination. The analysis was conducted utilizing the Wilcoxon signed-rank test within the SPSS version 22 statistical software. A statistical significance threshold of 0.05 was used to assess the importance of the results.

## 4. Results

### 4.1. Demographic Characteristics

This study found no notable differences in demographic characteristics between the two groups. All participants were enrolled in the same university, and their ages ranged from 21 to 26 years old. No significant difference was found in age (P = 0.386) or gender distribution (P = 0.895) between groups.

### 4.2. Primary Outcome: Gag Trigger Point Index

**Figure 2.**
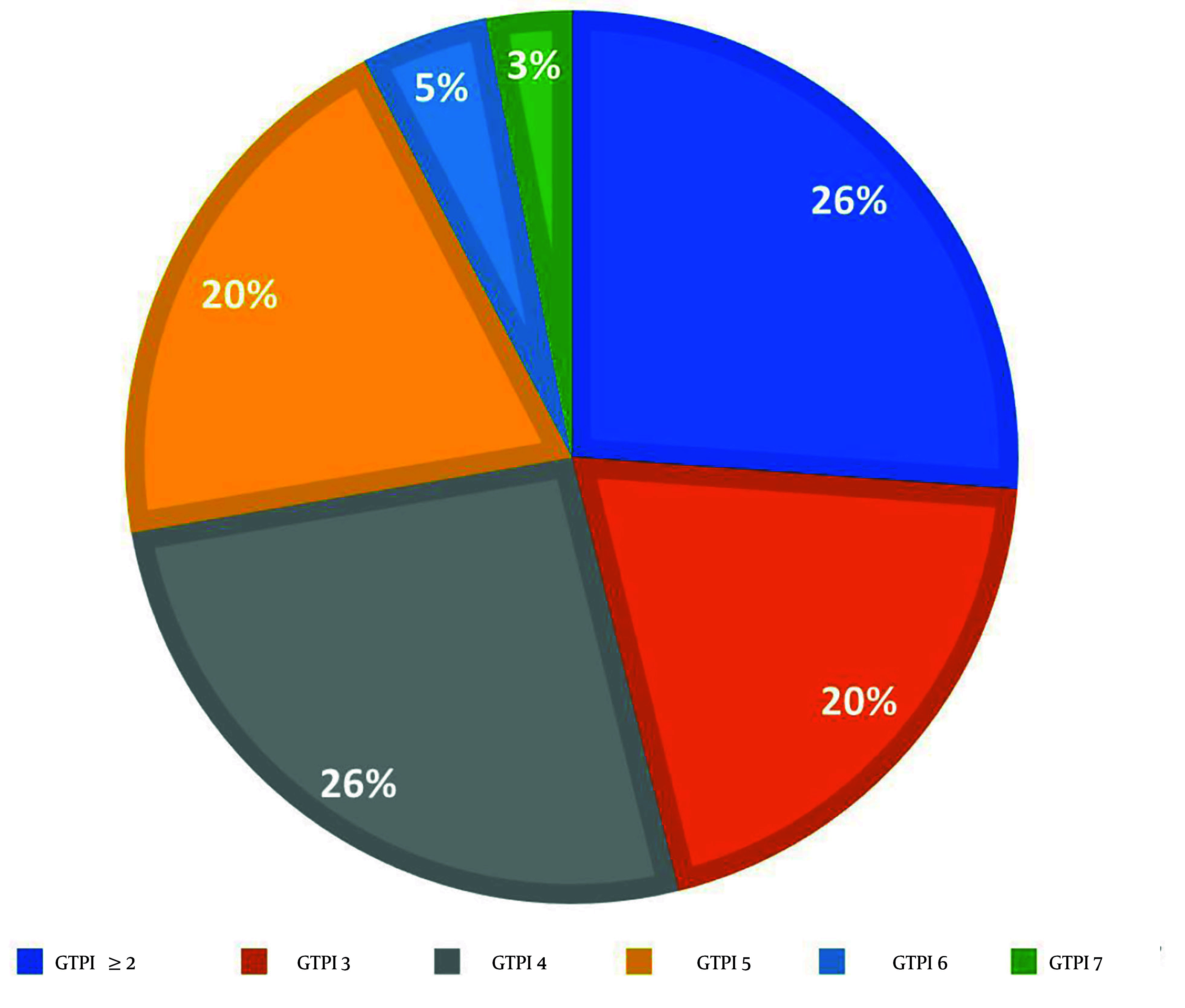
The distribution of participants based on their pre-intervention Gag Trigger Point Index (GTPI) scores

In relation to the GTPI test conducted prior to the intervention, the distribution of scores among participants was as follows: Seventeen, 13, 13, 3, and 2 participants scored 4, 5, 3, 6, and 7, respectively (Figure 2). Additionally, 17 volunteers exhibited a GTPI score of less than 2, leading to their exclusion from the study.

The results showed that before any intervention, mean ± SD GTPI scores were 4.04 ± 0.96 in the amitriptyline group and 4.46 ± 1.14 in the lidocaine group, with no significant difference (Mann-Whitney U = 229.5, Z = -1.35, P = 0.177, R = 0.20).

**Figure 3.**
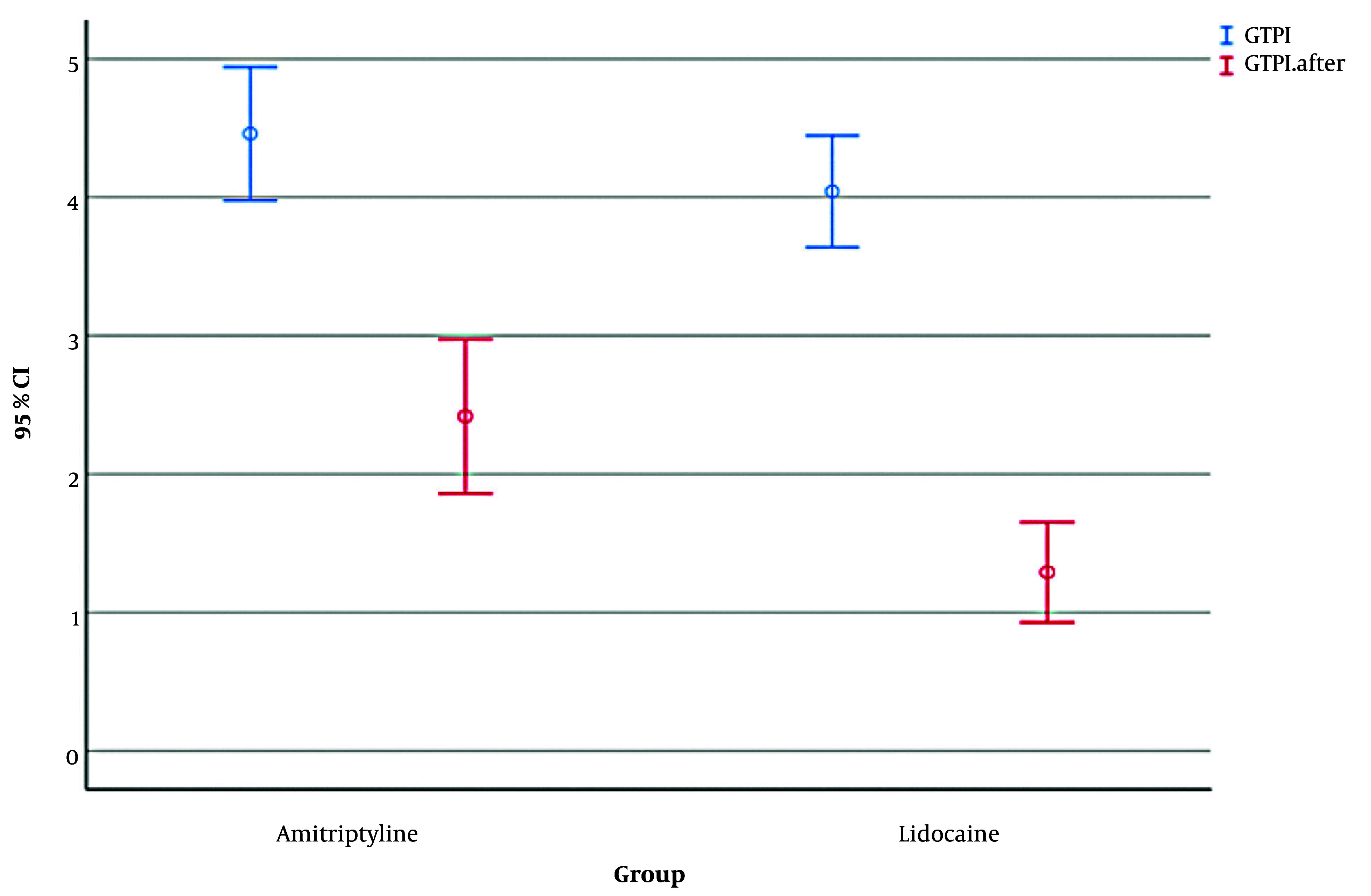
Error bar chart displaying the mean gag reflex before and after using amitriptyline mouthwash and lidocaine spray

Within-group changes revealed that both groups showed significant reductions in GTPI following intervention. In the amitriptyline group, GTPI decreased from 4.04 ± 0.96 to 1.29 ± 0.86 (Wilcoxon signed-rank Z = -4.20, P < 0.001, R = 0.61), and GTPI decreased from 4.46 ± 1.14 to 2.42 ± 1.32 in the lidocaine group (Z = -3.98, P < 0.001, R = 0.58). Between-group comparison of changes showed that although the mean reduction in GTPI was numerically larger in the amitriptyline group (Δ = -2.75 ± 0.96) than in the lidocaine group (Δ = -2.04 ± 1.17), this difference was not statistically significant (Mann-Whitney U = 240.0, Z = -1.76, P = 0.078, R = 0.20; Figure 3). 

### 4.3. Secondary Outcome: Patient Satisfaction with Taste and Smell

Regarding patient satisfaction with the taste and smell of the two medications, no significant differences were observed between groups in self-reported satisfaction with taste (Mann-Whitney U = 252, P = 0.081, R = 0.26) or smell (Mann-Whitney U = 360, P = 0.091, R = 0.25; Appendix 1 in Supplementary File).

## 5. Discussion

The study results indicated that both amitriptyline mouthwash and lidocaine spray were successful in reducing the intensity of the gag reflex in patients, with no statistically significant difference between their efficacy, taste, and smell. Various methods have been recommended to lessen the severity of the gag reflex, ranging from prescribing medications to employing non-pharmacological approaches (7, 23, 28). In 1977, Kramer and Braham (29) introduced the concept of using local anesthetics as a remedy for gagging issues, suggesting that patients may experience a reduced likelihood of gag reflex if the mucosal surfaces of the soft palate are desensitized. Amitriptyline, a tricyclic antidepressant, interacts with receptors near sodium channels in neurons, sharing receptor sites with local anesthetic agents. This interaction is distinct from its antidepressant effects and becomes apparent when amitriptyline is applied topically, particularly when it comes into contact with the oral mucosa (18). In 2018, Kakoei et al. (18) discovered that utilizing amitriptyline mouthwash resulted in local anesthetic effects for oral mucositis without causing systemic side effects. The reduction in pain severity observed with amitriptyline mouthwash exceeded that achieved with benzydamine hydrochloride mouthwash in their study.

Systemic antidepressant and anxiolytic effects of amitriptyline typically require days to weeks of administration (30). These systemic actions are mechanistically distinct from the immediate reduction in gag reflex observed in the present trial. The present results are therefore more plausibly attributed to local anesthetic-like receptor interactions rather than to its antidepressant or anxiolytic properties. However, the local pharmacology of topical amitriptyline remains insufficiently understood, and pharmacokinetic studies are needed to clarify whether its effect is mediated exclusively by local mucosal action or is partly influenced by systemic absorption.

While the causes of gagging are diverse and multifactorial, an exaggerated gag reflex can be linked to anxiety in some individuals (2, 31). Anxiety levels, ranging from mild to severe, can significantly contribute to an unpleasant and stressful dental experience (12). This is where amitriptyline might play a role in reducing the gag reflex in long-term consumption. Amitriptyline has been found effective in treating conditions such as anxiety, headaches, and insomnia (32). Its mechanism involves an increase in noradrenergic or serotonergic neurotransmission by blocking norepinephrine or serotonin transporters at presynaptic terminals. Prolonged use of amitriptyline leads to the desensitization of presynaptic autoreceptors and heteroreceptors, resulting in enduring alterations in monoaminergic neurotransmission (33).

Regrettably, there is a lack of studies analyzing the effects of amitriptyline on the gag reflex. Although studies such as Kakoei et al. (18) demonstrated its local anesthetic effect in oral mucositis, no study has analyzed its effects on the intensity of the gag reflex. There is an indication that amitriptyline may have utility in treating cyclic vomiting syndrome (CVS), characterized by recurrent, intense episodes of severe nausea and vomiting interspersed with periods of baseline health in over-5-year-old patients (34). It appears that low-dose amitriptyline is well-tolerated, and its prescription in gel and mouthwash forms poses no systemic adverse effects (18, 35). We found no side effects for both amitriptyline mouthwash and lidocaine spray in our participants. However, we only assessed analgesia during the immediate post-gargle period; no longer-term follow-up (hours or days later) was performed. Thus, the duration of pain relief or delayed side effects beyond the first few minutes is unknown for our study.

Lidocaine has been widely employed in previous studies to alleviate pain and reduce the gag reflex, with confirmed efficacy (23, 36-38). Nevertheless, the local application of lidocaine carries significant potential side effects. Utilizing lidocaine spray on the oral-pharyngeal cavity before intubation has been associated with a heightened frequency and severity of postoperative sore throat. The use of 10% lidocaine can cause mucosal damage due to solvent additives, such as menthol and ethanol, which may irritate the mucosa (39). Additionally, other potential side effects of lidocaine spray include nausea, vomiting, and dysphagia (40).

In light of the potential side effects associated with the local application of lidocaine, it is crucial to weigh the safety considerations when choosing an oral anesthetic for gagging control. Our study found no significant difference between amitriptyline mouthwash and lidocaine in reducing gag intensity, nor in their taste and smell from the patients' perspective. While this suggests that amitriptyline may lack distinct competitive advantages over lidocaine, it does emerge as a potential alternative for patients with allergies to lidocaine. This alternative may be particularly relevant given the observed side effects associated with lidocaine, providing clinicians with a valuable option in tailoring oral anesthesia to individual patient needs and sensitivities.

The main limitation of this study was the challenge of implementing a double-blind design. Because the two drugs were administered in different formulations, one as a spray and the other as a mouthwash, complete blinding was not feasible. In addition, systemic absorption was not assessed, leaving open the possibility of confounding between local and systemic effects. Other important limitations include the absence of pharmacokinetic evaluations, the small sample size, the lack of long-term follow-up, and the single-center setting. Therefore, future research should focus on large-scale, multicenter, double-blind trials with a follow-up period to better establish the safety, duration of action, and pharmacokinetic profile of topical amitriptyline.

The use of a fixed gargling duration and a single drug concentration may not capture the full range of therapeutic effects. It would be more informative in future studies to compare different gargling durations and concentrations (dilutions) to optimize mucosal absorption, analgesic efficacy, and patient tolerability.

### 5.1. Conclusions

The study's outcomes showed that amitriptyline may be considered a potential alternative to lidocaine spray for gag reflex management in dental patients, particularly those with lidocaine allergies. Further multicenter trials with larger samples, double-blinding, and pharmacokinetic analysis are needed to confirm these findings and determine the duration of action.

ijpr-24-1-160912-s001.pdf

## Data Availability

The dataset presented in the study is available on request from the corresponding author during submission or after publication.

## Clinical Trial Registration Code:

This trial is registered in the Iranian Registry of Clinical Trials (IRCT) with the identifier IRCT20190608043843N1.
