# Supplementary material for: Exploring the Role of Amitriptyline in Modulating Gag Reflex Sensitivity
Source: Iran J Pharm Res. 2025 Oct 15;24(1):e160912. doi: 10.5812/ijpr-160912 (PMC12552848; doi:10.5812/ijpr-160912)
Supplement: ijpr-24-1-160912-s001.pdf [file ijpr-24-1-160912-s001.pdf]

Appendix 1: The level of medications' taste and smell satisfaction

	Taste		Smell	
Drug Groups / Intensity	Amitriptyline	Lidocaine spray	Amitriptyline	Lidocaine spray
**Good**	0	0	2	0
**Moderate**	0	3	15	12
**Weak**	24	21	7	12
**P value**	0.074		0.089	
